# Correcting overestimation of approximate traditional reliabilities with herd-sire interactions when young genomic bulls are used in few herds

**DOI:** 10.1186/s12711-025-00984-0

**Published:** 2025-06-23

**Authors:** Joe-Menwer Tabet, Ignacio Aguilar, Matias Bermann, Daniela Lourenco, Ignacy Misztal, Paul M. VanRaden, Zulma G. Vitezica, Andres Legarra

**Affiliations:** 1 Department of Animal and Dairy Science, University of Georgia, Athens, GA 30602 USA; 2 Council on Dairy Cattle Breeding, Bowie, MD 20716 USA; 3 Instituto Nacional de Investigación Agropecuaria, 11500 Montevideo, Uruguay; 4 U.S. Department of Agriculture, Agricultural Research Service, Animal Genomics and Improvement Laboratory, Beltsville, MD 20705-2350 USA; 5 GenPhySE, INPT, INRAE, ENVT, 31326 Castanet Tolosan, France

## Abstract

**Background:**

Differential treatment of daughters of the same sire within a herd is modelled as the herd-sire effect. Recent changes in management practices may have led to the extensive use of certain bulls in a limited number of herds. In that case, although the effect can be well accounted for in genetic evaluation models, some approximation methods for reliabilities do not consider it correctly, leading to an overestimation of some sires’ approximated reliabilities. This study assessed the potential bias of these approximated reliabilities due to the herd-sire effect in both simulated and real dairy cattle records. Two existing methods were tested: Misztal–Wiggans, which includes a specific modification for herd-sire, and Tier–Meyer, which does not. We also modified and tested a Tier–Meyer method considering the herd-sire effect.

**Results:**

We observed that in the presence of the herd-sire effect, reliabilities obtained by approximations were overestimated by the Tier–Meyer method for sires with many daughters in a limited number of herds. This was true even for sires with a large number of daughters. The Misztal–Wiggans method performed correctly. We introduced a modified Tier–Meyer method that weighs the information transmitted by the daughter to the sire as a function of the herd-sire information. As a result, the modified Tier–Meyer method performed well in both simulated and real data. For cows, the inclusion of the herd-sire effect had minimal impact.

**Conclusions:**

This study identified possible overestimation of approximated reliabilities of sires with daughters concentrated in a few herds when there is a herd-sire effect. This bias occurs when the herd-sire effect is not correctly modeled in reliability approximation methods. Methods that specifically accounted for the herd-sire effect produced unbiased reliability estimates.

## Background

Over the past two decades, the number of U.S. dairy farms has decreased while the number of large farms has grown. For example, farms with fewer than 1000 cows have declined, while those with 1000 or more cows have increased [1]. These large herds tend to have systematic management practices, often using a few bulls to simplify their operations. Alongside this trend, genomic selection has led to the increased use of young genomic bulls, which have limited semen straw availability. As a result, some young bulls are exclusively used in a small number of herds instead of the traditional sampling approach (e.g., common around the year 2000) in which sires were more widely assigned across herds. Moreover, the extensive use of a single sire within a herd can result in preferential treatment of that sire’s daughters. This can be modelled as a (random) herd-sire (HS) effect or, equivalently, as creating an environmental covariance between half-sibs of the same sire within a herd.

Historically, the importance of the HS effect was greater before the widespread use of Artificial Insemination (AI) because natural service sires have daughters only in one herd, resulting in higher environmental covariance. In the traditional sampling approach, AI sires had several offspring distributed in many herds [2, 3]. The HS effect has recently regained importance with the recent changes in management practices in large herds, leading to the non-random, less distributed allocation of sires to herds [4].

The HS effect is often introduced in the genetic evaluation model [5–7]. Failing to do so can introduce bias and overestimate reliabilities [8]. Additionally, Banos and Shook [9] noted that ignoring the HS effect overestimates the prediction and accuracy of sire evaluations, particularly when sires have daughters in a few herds. Previous reliability approximation methods include specific corrections of reliabilities for herd-sire interactions [5–8].

The overestimation of reliabilities by approximation methods when there is an HS effect is a problem specific to HS, unlike, say, contemporary groups (CG) [10–12]. This bias in reliabilities is shown in the following example. Consider a “sire model” with two sires and two herds, each with 100 records. Assume there is no herd effect, but there is a preferential treatment HS effect, i.e., daughters of sire 1 are treated differently than daughters of sire 2, with these differences varying by herd. If each sire is assigned to a single herd, separating the sire from the preferential treatment captured by HS becomes impossible, i.e., they are partially confounded. Conventional approaches for reliability, e.g. [12], rely on the number of records of its HS effect (e.g., 100); however, the confounding of the HS (an environmental effect) and the sire itself (a genetic effect) is not addressed. If each sire is assigned to both herds, then there are two HS effects per sire; there is no confounding, and reliability increases with the number of records in the CG.

The genetic evaluation model used by the Council on Dairy Cattle Breeding (CDCB; Bowie, MD) for U.S. dairy cattle includes the HS effect for most traits. However, the estimated reliabilities are an approximation that ignores the off-diagonals that associate HS with sires of daughters within the same herd and does not consider whether individuals in the same contemporary group are related. So far, recent reliability estimation methods have not addressed the overestimation of reliabilities due to the HS effect. As a result, there are anecdotal reports of genomic bulls assigned to a few herds whose traditional (non-genomic) reliabilities may be overestimated. For example, in December 2024, the traditional estimated breeding values (EBVs) for milk yield of bulls JEUSA000117723269 and JE840003012659218 had reliabilities of 0.97 and 0.98, respectively, according to the CDCB. These reliabilities were based on 377 daughters in a single herd for the first bull and 390 daughters across three herds for the second. These reliabilities are likely overestimated because the model includes HS interaction. Although such cases represent a small fraction of the population, they often involve significant financial investments for their owners. Therefore, it is fair that they should receive unbiased reliability.

The objective of this study was to explore, through simulation and real data, the extent of bias in reliability approximation when the HS effect is included in the model and ignored in the approximation process and provide possible solutions to the bias using approximate methods. Two approximation methods were investigated. Misztal and Wiggans [13] proposed a method for approximating PEV by simplifying computations, but its applicability is limited in complex models. Tier and Meyer [10] introduced a three-step reliability approximation method adaptable to multi-trait and complex models, but it overlooks the HS effect, potentially causing overestimation. As a byproduct, a modification to the approach by Tier and Meyer [10] is proposed to account for the HS effect.

## Methods

### Methods to estimate reliabilities

#### Existing methods

The reliability of the EBV for the ith animal $\left(EB{V}_{i}\right)$ can be directly calculated by inverting the coefficient matrix of the Mixed Model Equations (MME) as $RE{L}_{EXAC{T}_{i}}=1-\frac{PE{V}_{i}}{{a}_{ii}{\sigma }_{u}^{2}}$, where $PE{V}_{i}$ is the prediction error variance of $EB{V}_{i}$, obtained from the diagonal of the inverse of the MME, ${a}_{ii}$ is the diagonal element in the numerator relationship matrix $\left(\mathbf{A}\right)$ for animal $i$, and ${\sigma }_{u}^{2}$ is the additive genetic variance. The computational costs of inversion are too high for large data sets. Methods have been developed to approximate PEV, either by approximating the diagonal elements of the inverse coefficient matrix [13, 14] or by sequentially accumulating information from the animal’s own or progeny records, parents, or other relatives [10].

Misztal and Wiggans [13] proposed a method that approximates PEV by ignoring off-diagonals and absorbing the non-genetic model effects (i.e., subclasses, permanent environmental effect (pe)). This method was further modified to account for the HS effect in the reliability estimate by using a weighting factor based on the expected reliability of a sire, which accounts for the number of daughters within each herd-sire subclass [5]. This results in an iterative method that loops through the pedigree while considering this weighting factor. These “herd-sire weights” accumulate daughter weights across herds in which a sire has daughters while accounting for the information from each lactation separately and adjusting for the CG effects. However, a limitation of the method in [13] is that extending to more complex models (e.g., random regression models) and complex missing trait patterns is hard because its computation is single-trait based and embedded inside a loop going through the pedigree, as shown in the Appendix of [13]. Their approach, along with a method to consider genomic information in the approximation [15], is programmed in the software accf90GS2 from the BLUPF90 suite [16]. We call this approximation of reliability $R{EL}_{MW}$.

Tier and Meyer [10] developed a three-step approach to approximate PEV by adjusting the diagonal blocks of the coefficient matrix. The first adjustment accounts for the animal’s records based on the size of its subclass, followed by accumulating information on progeny and other descendants and, finally, information on parents, ancestors, and other relatives. The advantage of this method is its natural extension to multiple trait and random regression models. However, it does not explicitly account for the HS effect and a similar modification, as used in $RE{L}_{MW}$ would be needed to incorporate the HS effect properly. This method is programmed in the software accf90GS3 from the BLUPF90 suite (still unreleased). We call this approximation of reliability $R{EL}_{TMorig}$.

#### Modified Tier–Meyer to consider herd-sire effect

The original Tier–Meyer method does not account for the HS effect in its current form. This method includes a modification to adjust for contemporary groups with a high proportion of half-sibs, e.g., paddocks sired by a single bull (which may occur e.g., in beef cattle). However, this differs from our problem, where a herd can have a diverse set of sires, and the herd effect be correctly estimated, yet still, the herd-sire effect exists, e.g. each group of half sibs is treated differently. Even in large herds, if sires are not well distributed across herds, the reliability will be overestimated. For instance, consider a herd with 1000 cows, 100 of which are daughters of a single sire, and these are the sire’s only daughters. Although the herd effect would be correctly estimated, the HS effect would be confounded with the sire effect. When calculating reliabilities using only the CG adjustment (excluding the herd-sire modification), we observed minor changes in reliability values, similar to those obtained with $RE{L}_{TMorig}$.

In this study, we propose a modification to incorporate it. This modification, referred to as $RE{L}_{TMnew}$, involves assigning a weight to the matrix ${\mathbf{E}}_{\text{l}}$ of Eq. (6) of Tier and Meyer [10], rather than absorbing the effect as is done with pe and fixed effects. Absorbing the HS effect would create off-diagonal elements connecting sires and herd sires, and ignoring those off-diagonals results in wrong values of reliabilities. This equation sends contributions of information from offspring to parents, so the adjusted equation for animal $i$ and its $l=1\dots {p}_{i}$ progeny is:

1 $${\mathbf{E}}_{i}=\frac{1}{3}{\mathbf{G}}_{0}^{-1}-\frac{4}{9}{\mathbf{G}}_{0}^{-1}{\left({\mathbf{D}}_{i}+{\sum }_{l=1}^{{p}_{i}}{\mathbf{K}}_{l}{\mathbf{E}}_{l}{\mathbf{K}}_{l}+\frac{4}{3}{\mathbf{G}}_{0}^{-1} \right)}^{-1}{\mathbf{G}}_{0}^{-1}$$

where ${\mathbf{G}}_{0}$ is a matrix with genetic covariances and $\mathbf{D}$ and $\mathbf{E}$ are submatrices corresponding, respectively, to the contribution of the animal's own phenotype (after absorbing the fixed and permanent environment effects) and to the contribution of its progeny. Matrix ${\mathbf{K}}_{l}$ is a (number of traits by number of traits) diagonal matrix, different for each animal $l$, with its $\left(k,k\right)$ element equal to $\sqrt{{c}_{l(k)}}$. The weight ${c}_{l(k)}$ represents the amount of information cow $l$ for trait $k$ gives to her sire across potentially different herds (i.e., if the cow changed herds) compared to a weight of 1 if there was no HS effect. In the beginning, we compute weights within specific herds, and then we average them. A weight is calculated for all cows within each herd-sire $j$ for trait $k$ as: $ch{s}_{j(k)}=\frac{{EDC}_{j(k)}}{\sum {w}_{j(k)}}$. This represents how much information one cow contributes within the same herd-sire subclass relative to an evaluation model where herd-sire has no effect. The denominator $\sum {w}_{j(k)}$ is the weighted sum of the number of records for trait $k$. In many cases (at least in the US dairy evaluation), records are weighted (e.g., by the length of lactation [6] or the number of inseminations [17]), and hence the notation $\sum {w}_{j(k)}$. In unweighted evaluations $\sum {w}_{j(k)}$ is simply the number of records. These weights ${w}_{j(k)}$ represent amount of information or, in statistical terms, they are used to scale residual variances, and they are *not* the weights ${c}_{l(k)}$ above. The term $ED{C}_{j(k)}$ represents the total Effective Daughter Contributions ($EDC$) present in herd-sire $j$ for trait $k$. We use $EDC$ instead of Effective Record Contributions ($ERC$) because, for sire reliability purposes, 1 cow equals 1 $EDC$, and the algebra in [6] is conceived for $EDC$ s. This $ED{C}_{j(k)}$ is computed separately for each trait in two steps. First, the formula by VanRaden and Wiggans [6] gives the maximum possible reliability of a sire due to information contributed by daughters located in a single herd-sire $j$ for trait $k$, $RE{L}_{ani{m}_{j\left(k\right)}}^{*}$:

2 $$RE{L}_{ani{m}_{j\left(k\right)}}^{*}=\frac{{\sigma }_{s}^{2}}{{\sigma }_{s}^{2}+{\sigma }_{hs}^{2}+\frac{{\sigma }_{\epsilon }^{2}}{\sum {w}_{j(k)}}}$$

where, for trait$k$, ${\sigma }_{s}^{2}=0.25{\sigma }_{u}^{2}$ is the variance component of the sire effect; ${\sigma }_{hs}^{2}$ is the variance component of the HS effect; ${\sigma }_{\epsilon }^{2}={\sigma }_{e}^{2}+{0.5\sigma }_{u}^{2}$, assuming the dam’s breeding value is known [6]. If permanent environment (pe) effects are included, then${\sigma }_{\epsilon }^{2}= {\sigma }_{e}^{2}+ {0.5\sigma }_{u}^{2}+{\sigma }_{pe}^{2}$. Finally, $\sum {w}_{j(k)}$ is as defined above. Second, this quantity is transformed into $EDC$ [6]:

3 $$ED{C}_{j\left(k\right)}=\frac{4-{2h}^{2}}{{h}^{2}}\left(\frac{RE{L}_{ani{m}_{j\left(k\right)}}^{*}}{1-RE{L}_{ani{m}_{j\left(k\right)})}^{*}}\right)$$

This represents the effective number of daughters a particular herd-sire contains, including the herd-sire effect in the model. Compared to $\sum {w}_{j(k)}$, this indicates how much useful information is present in the herd-sire subclass. Thus, to obtain a weight for each cow in the herd-sire subclass, $ED{C}_{j(k)}$ is divided by $\sum {w}_{j(k)}$ to obtain $ch{s}_{j(k)}=\frac{{EDC}_{j(k)}}{\sum {w}_{j(k)}}$, which is equal for all cows in the herd-sire subclass. However, because a cow can potentially change herds across lactations, weights need to be defined per cow, accounting for all herds to which the cow was assigned to. The final weight ${c}_{l(k)}$ for cow $l$ in trait $k$ is an average of the weights $ch{s}_{j(k)}$ of all the herd-sires $j$ in which the cow $l$ participated for that trait, i.e., if the cow had one lactation in herd A and two lactations in herd B, ${c}_{l(k)}=\frac{1\times ch{s}_{A\left(k\right)}+2\times ch{s}_{B\left(k\right)}}{3}$.

Finally, the weights are not used when transmitting the information from parents to offspring (Eq. (7) in [10]) because they consider the phenotypic information, which is already done in our Eq. (1).

### Data and model

All data came from simulated or preexisting databases. Ethical approval for the use of animals was thus deemed unnecessary.

#### Simulated data

**Fig. 1:**
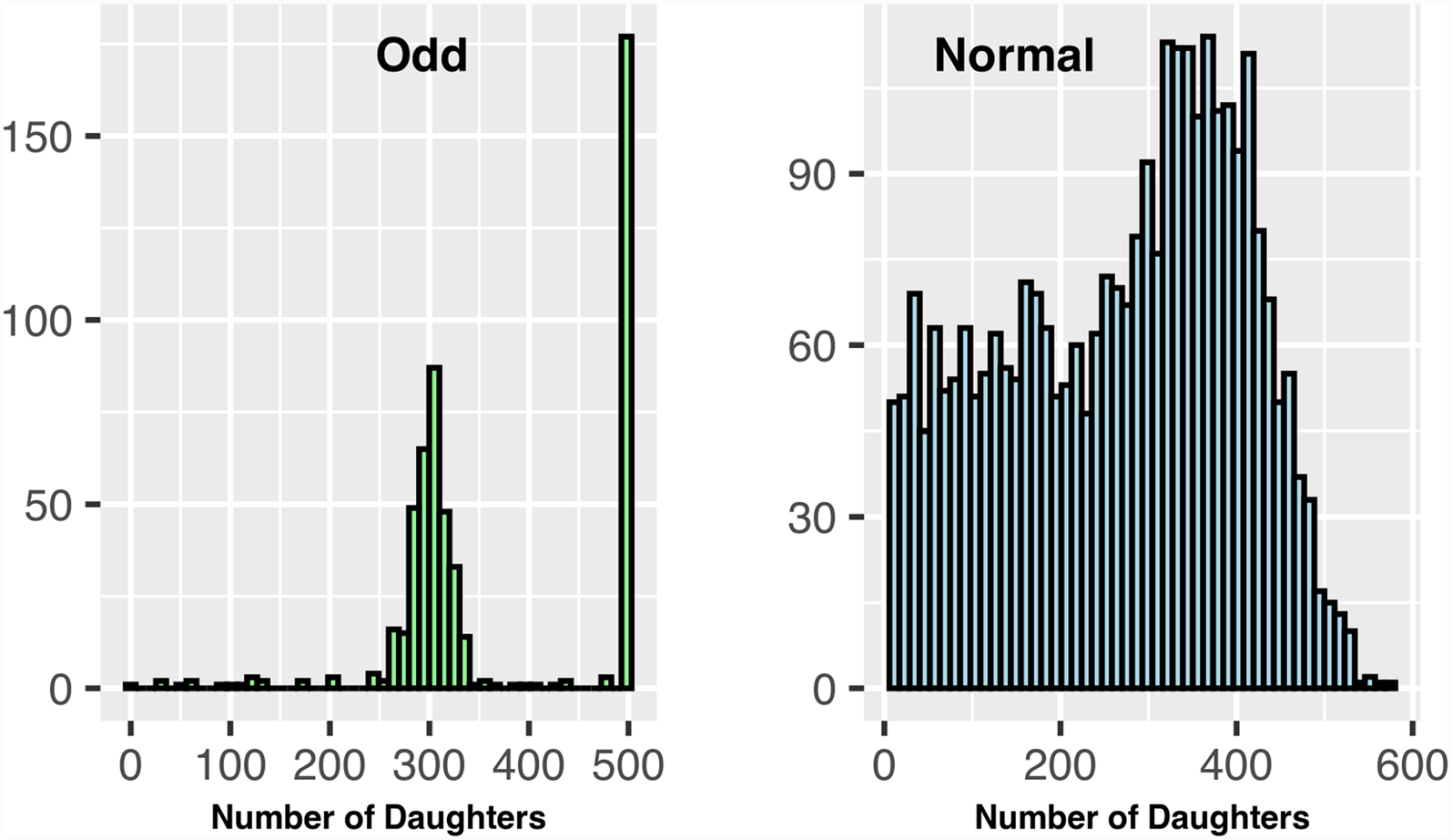
Distribution of daughters per sire. Odd: Sires with many daughters concentrated in a small number of herds; Normal: Sires with daughters more evenly distributed across many herds

**Fig. 2:**
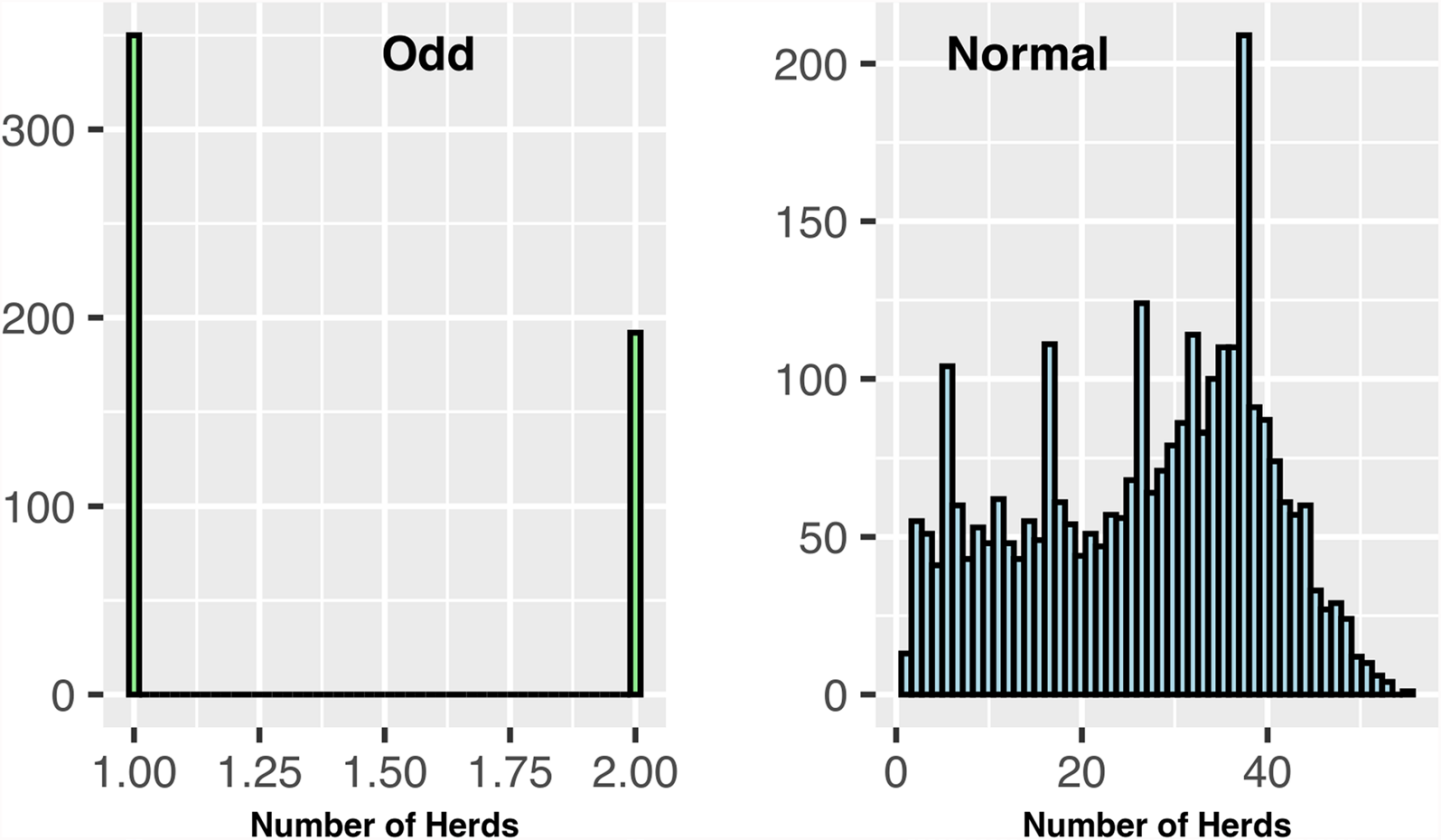
Distribution of herds per sire. Odd: Sires with many daughters concentrated in a small number of herds; Normal: Sires with daughters more evenly distributed across many herds

To examine changes in approximated reliabilities due to ignoring the HS effect, we conducted a simulation that generates a data structure mimicking a two-generation U.S. dairy cattle population -parent and offspring- with sires classified into “Odd” and “Normal.” Odd sires have a limit of 500 straws, whose use was deemed in large “quotas” of a few hundred straws by the same producers at a time; as a result, they were limited to 1–2 herds, with a maximum of 500 daughters. Normal sires had 10–600 daughters spread across 1–60 herds. We simulated two types of herds: “Large” with more than 3000 animals and “Average” with less than 3000. A total of 3542 sires (542 Odd and 3000 Normal) and 1000 herds were simulated. All daughters had a unique dam and a single record. As a result, we created a structure consisting of sires with varying reliabilities based on the type and distribution of daughters. Figures 1 and 2 illustrate the distribution of daughters and herds per each sire type.

We considered a single-trait animal model with the herd as a fixed effect, HS effect with variance $\mathbf{I}{\sigma }_{hs}^{2}$, where ${\sigma }_{hs}^{2}=$ 5, an animal additive genetic effect with a mean zero and variance $\mathbf{A}{\sigma }_{u}^{2}$ with ${\sigma }_{u}^{2}=$ 20. The residual variance was 75. Reliabilities were obtained either from the inverse of the MME ($RE{L}_{EXACT}$) or from the approximation methods Misztal–Wiggans ($RE{L}_{MW}$), which corrects for herd and herd-sire, original Tier–Meyer ($RE{L}_{TMnorig}$), which corrects for herd only, and modified Tier–Meyer ($RE{L}_{TMnew}$), which corrects for herd and herd-sire.

#### Real data

We also used data provided by the CDCB. A subset of records for Jersey (JE) animals was extracted. The first data included 7,824,163 milk yield records from 2,967,998 cows, 3,863,135 animals in the pedigree, and 687,889 HS; this will be referred to as DataFull. However, this file proved too large to obtain exact reliabilities by inversion, which we attribute to a large number of fill-ins in the sparse Cholesky decomposition caused by the HS effect. Thus, we further reduced the dataset to records of cows born after 2015, resulting in DataReduced, with 1,605,821 records, 776,390 cows in records, 1,725,100 animals in the pedigree, and 88,035 HS. In both cases, the pedigree spanned as many ancestors as possible.

We ran a single-trait repeatability model with CG and age-parity as fixed effects. The HS (${\sigma }_{hs}^{2}=7.3\times {10}^{6}$) and animal additive genetic (${\sigma }_{u}^{2}=2.1\times {10}^{7}$) effects were modeled as random effects defined earlier, with pe random effect with a mean zero and variance ${\sigma }_{pe}^{2}$, where ${\sigma }_{pe}^{2}=2.9 \times {10}^{7}$ the variance component of the pe effect. The residual variance was $4.7 \times {10}^{7}$. Computation of reliabilities ignored Unknown Parent Groups in all cases, as typically assumed.

Exact reliabilities $RE{L}_{EXACT}$ were obtained using blupf90+, $R{EL}_{MW}$ are computed by accf90GS2, and $RE{L}_{TMorig}$ and $RE{L}_{TMnew}$ are computed by accf90GS3, all programs from the BLUPF90 suite [16].

### Reliability comparison

For the simulated dataset and DataReduced, we compared $RE{L}_{EXACT}$ versus $RE{L}_{MW}$, $RE{L}_{TMnew}$ and $RE{L}_{TMorig}$. In the case of DataFull, $RE{L}_{EXACT}$ was not available because it was impossible to invert the MME due to their large size, so we compared $RE{L}_{MW}$ versus $RE{L}_{TMnew}$ and $RE{L}_{TMorig}$.

The comparisons were detailed by bull category. In the simulated data, bulls are classified as “Odd” and “Normal”. With the real JE data, it is necessary to identify sires that may have many daughters concentrated in only a few herds to categorize them as “Odd” or “Normal”. In that case, we calculated a measure that we called the h-index, analogous to the Google Scholar h-index [18]. A sire with an h-index of $n$ has at least $n$ daughters in $n$ herds. For instance, a sire with a daughter count of 10, 7, 4, and 2 across 4 herds has an h-index of 3 as it has at least 3 daughters in 3 herds. Sires with many daughters concentrated only in a few herds were classified as “Odd” with an h-index ≤ 5. In contrast, sires with a more even distribution of daughters across multiple herds were classified as “Normal” if they had an h-index ≥ 10, implying at least 100 daughters with records. Results for sires between h-indexes of 5 and 10 were inconclusive in preliminary inspection, and we did not use them for diagnostics. For example, consider two sires, each with 200 daughters. Sire 1 has 100, 50, 23, 20, 3, 2, 1, and 1, daughters across 8 herds, giving an h-index of 4. This indicates that most of its daughters are concentrated in a few herds, resulting in a larger number of half-siblings within each herd. Sire 2, on the other hand, has 15 daughters in each of 11 herds and 5 daughters in each of another 7 herds, resulting in an h-index of 11, leading to less influence of preferential treatment in the sire proof.

## Results and discussion

**Fig. 3:**
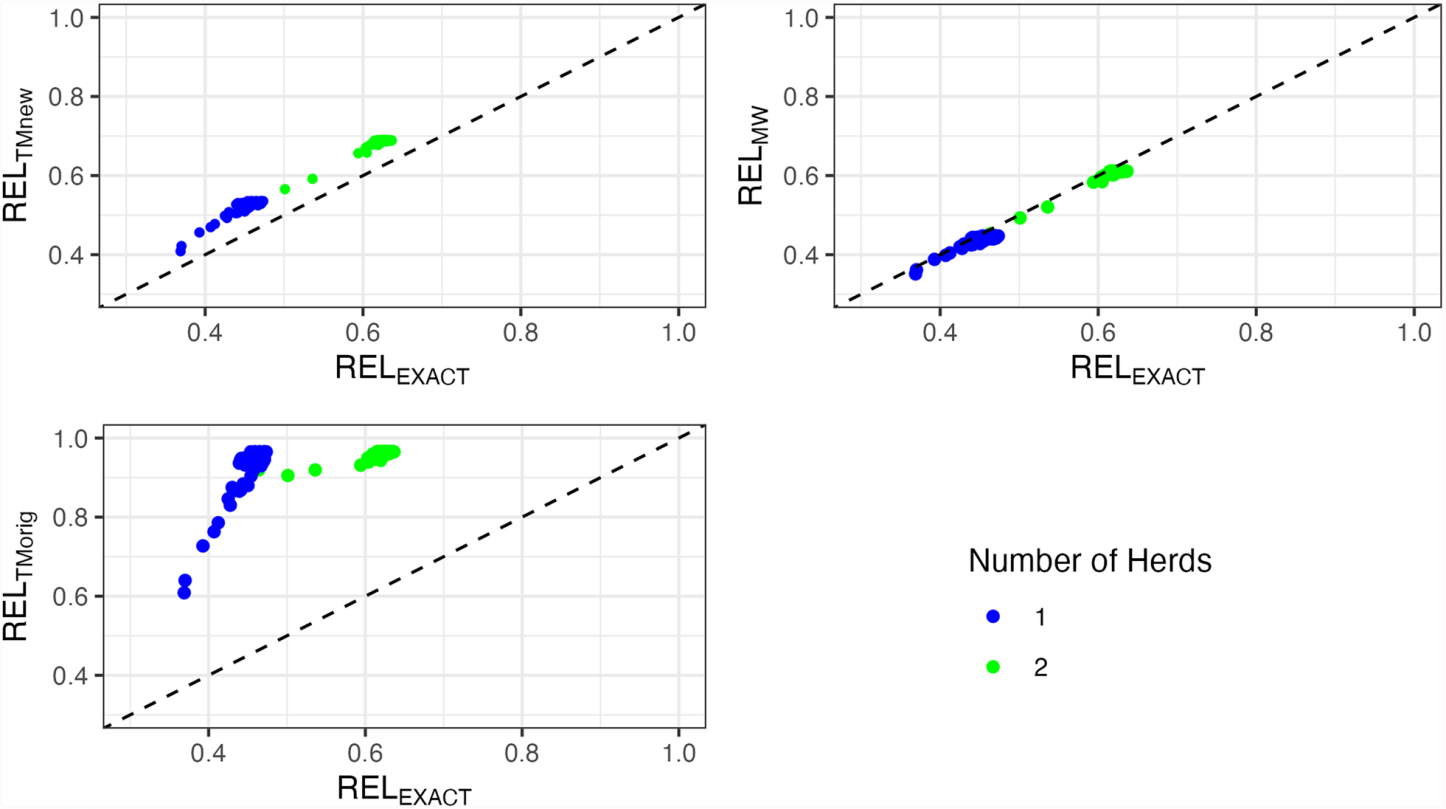
Reliabilities of Odd sires from the simulated dataset. Odd: Sires with many daughters concentrated in a small number of herds; Normal: Sires with daughters more evenly distributed across many herds; REL_EXACT_: Exact reliabilities from the inverse of the coefficient matrix with blupf90+; REL_MW_: Approximated reliabilities based on Misztal–Wiggans; REL_TMnew_: Approximated reliabilities based on modified Tier–Meyer; REL_TMorig_: Approximated reliabilities based on original Tier–Meyer

For simulated data, Fig. 3 and Table 1 display the results of the true and approximated reliabilities of Odd bulls. Both $RE{L}_{MW}$ and $RE{L}_{TMnew}$ correctly estimated reliabilities with a slight overestimation for $RE{L}_{TMnew}$, whereas $RE{L}_{TMorig}$ overestimated reliabilities by more than 0.40 points because this method does not explicitly account for the HS effect. Table 1 presents the correlations and slope values of the regression of $RE{L}_{EXACT}$ on the approximated reliabilities. The correlations were 0.99 for both $RE{L}_{MW}$ and $RE{L}_{TMnew}$ and 0.48 for $RE{L}_{TMorig}$. Including the HS in the Tier–Meyer algorithm, the correlations increased. Additionally, the modified Tier–Meyer improved the slope of the regression of $RE{L}_{EXACT}$ on approximated reliabilities from 0.85 ($RE{L}_{TMorig}$) to 1.03 ($RE{L}_{TMnew}$).

**Table 1:** Reliability means, correlation, and regression of REL_EXACT_ on approximated reliabilities of Odd and Normal sires from simulated dataset

	Odd^a^			Normal		
	Mean	b_1_	Correlation	Mean	b_1_	Correlation
REL_EXACT_^b^	0.51			0.86		
REL_MW_	0.50	1.00	0.99	0.85	0.97	0.99
REL_TMnew_	0.58	1.03	0.99	0.87	1.04	0.99
REL_TMorig_	0.95	0.85	0.48	0.90	1.22	0.99

^a^Odd: Sires with many daughters concentrated in a small number of herds (N = 542); Normal: Sires with daughters more evenly distributed across many herds (N = 3000)

^b^REL_EXACT_: Exact reliabilities from the inverse of the coefficient matrix with blupf90 + ; REL_MW_: Approximated reliabilities based on Misztal–Wiggans; REL_TMnew_: Approximated reliabilities based on modified Tier–Meyer; REL_TMorig_: Approximated reliabilities based on original Tier–Meyer

Table 1 shows Normal sires' true and approximated reliabilities. We can see a slight overestimation with $RE{L}_{TMnew}$. We see correlations of 0.99 across all three cases. This shows that when sires are well spread among herds, the correction for HS is less important. However, $RE{L}_{TMorig}$ (${b}_{1}=$ 1.22) were under-dispersed compared to $RE{L}_{TMnew}$ and $RE{L}_{MW}$ with ${b}_{1}$ values close to 1.

$RE{L}_{TMnew}$ slightly overestimated reliabilities, likely due to only accounting for first-order relationships (i.e., parent-progeny). This limitation signifies that more distant descendants within the same subclass (e.g., several generations of descendants in the same contemporary) are still implicitly treated as informative. As a result, the method overestimates the actual amount of information available, leading to overestimated reliabilities [10].

**Fig. 4:**
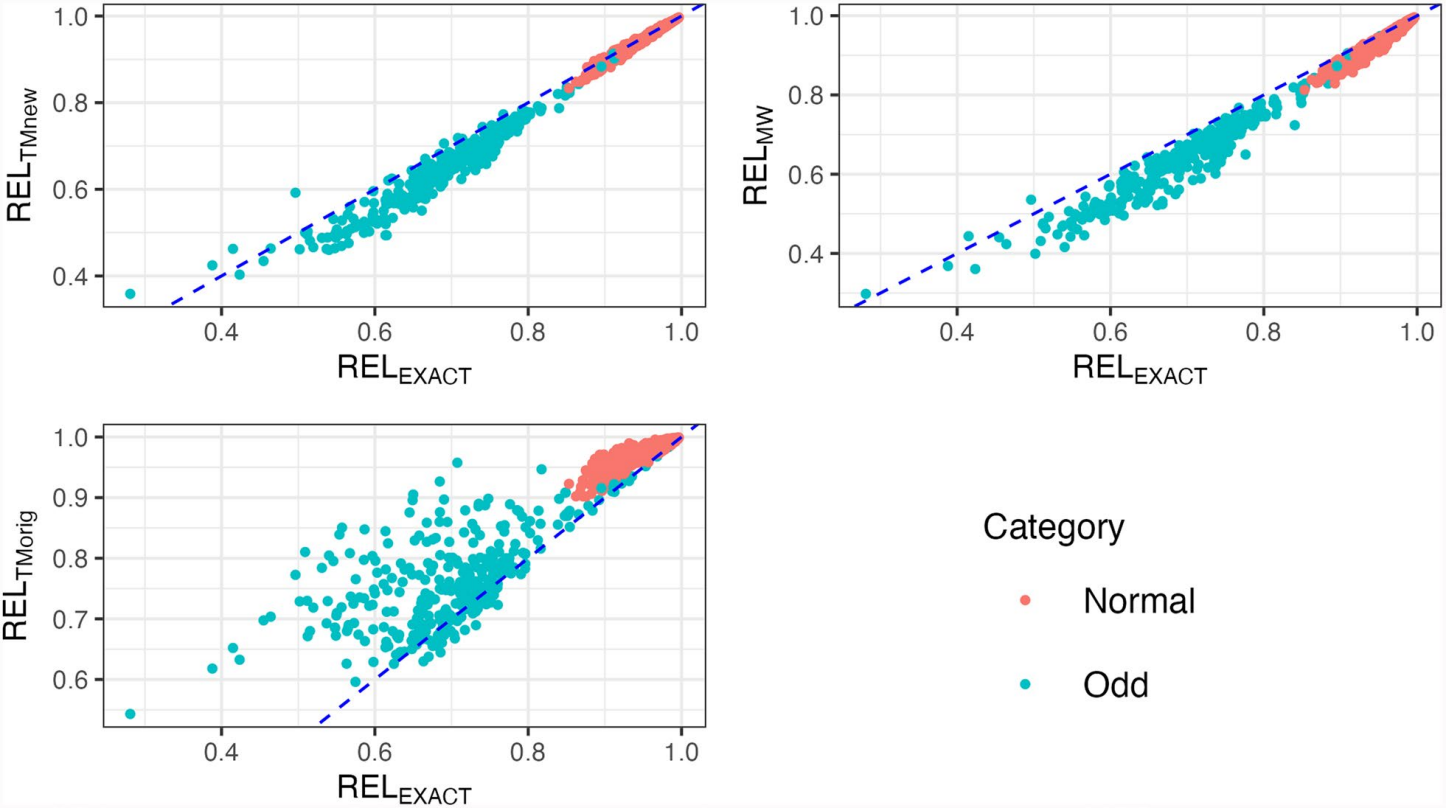
Reliabilities of Odd and Normal sires from DataReduced. Odd: Sires with many daughters concentrated in a small number of herds; Normal: Sires with daughters more evenly distributed across many herds; REL_EXACT_: Exact reliabilities from the inverse of the coefficient matrix with blupf90+; REL_MW_: Approximated reliabilities based on Misztal–Wiggans; REL_TMnew_: Approximated reliabilities based on modified Tier–Meyer; REL_TMorig_: Approximated reliabilities based on original Tier–Meyer

For DataReduced, results in Fig. 4 and Table 2 show exact and approximated reliabilities for Odd and Normal sires according to the h-index; other sires are excluded from the figure for clarity. Approximated reliabilities $RE{L}_{TMnew}$ and $RE{L}_{MW}$ were only slightly under-estimated for Odd sires, whereas $RE{L}_{TMorig}$ clearly over-estimated reliabilities. The correlations and slopes of $RE{L}_{EXACT}$ regressed on $RE{L}_{TMnew}$ or $RE{L}_{MW}$ were close to 1, while $RE{L}_{TMorig}$ showed lower correlation and higher dispersion. For Normal sires, all approximated reliabilities were quite similar to $RE{L}_{EXACT}$, except for the slopes. Again, we confirm that for Odd sires, the correction of reliabilities for HS is important, but less so for Normal sires.

**Table 2:** Reliability means correlation and regression of REL_EXACT_ on approximated reliabilities of Odd and Normal sires from DataReduced

	Odd^a^			Normal		
	Mean	b_1_	Correlation	Mean	b_1_	Correlation
REL_EXACT_^b^	0.70			0.95		
REL_MW_	0.65	0.86	0.97	0.98	0.77	0.98
REL_TMnew_	0.66	0.90	0.97	0.95	0.89	0.99
REL_TMorig_	0.76	0.79	0.64	0.97	1.36	0.90

^a^Odd: Sires with many daughters concentrated in a small number of herds (N = 344); Normal: Sires with daughters more evenly distributed across many herds (N = 544)

^b^REL_EXACT_: Exact reliabilities from the inverse of the coefficient matrix with blupf90+; REL_MW_: Approximated reliabilities based on Misztal–Wiggans; REL_TMnew_: Approximated reliabilities based on modified Tier–Meyer; REL_TMorig_: Approximated reliabilities based on standard Tier–Meyer

**Fig. 5:**
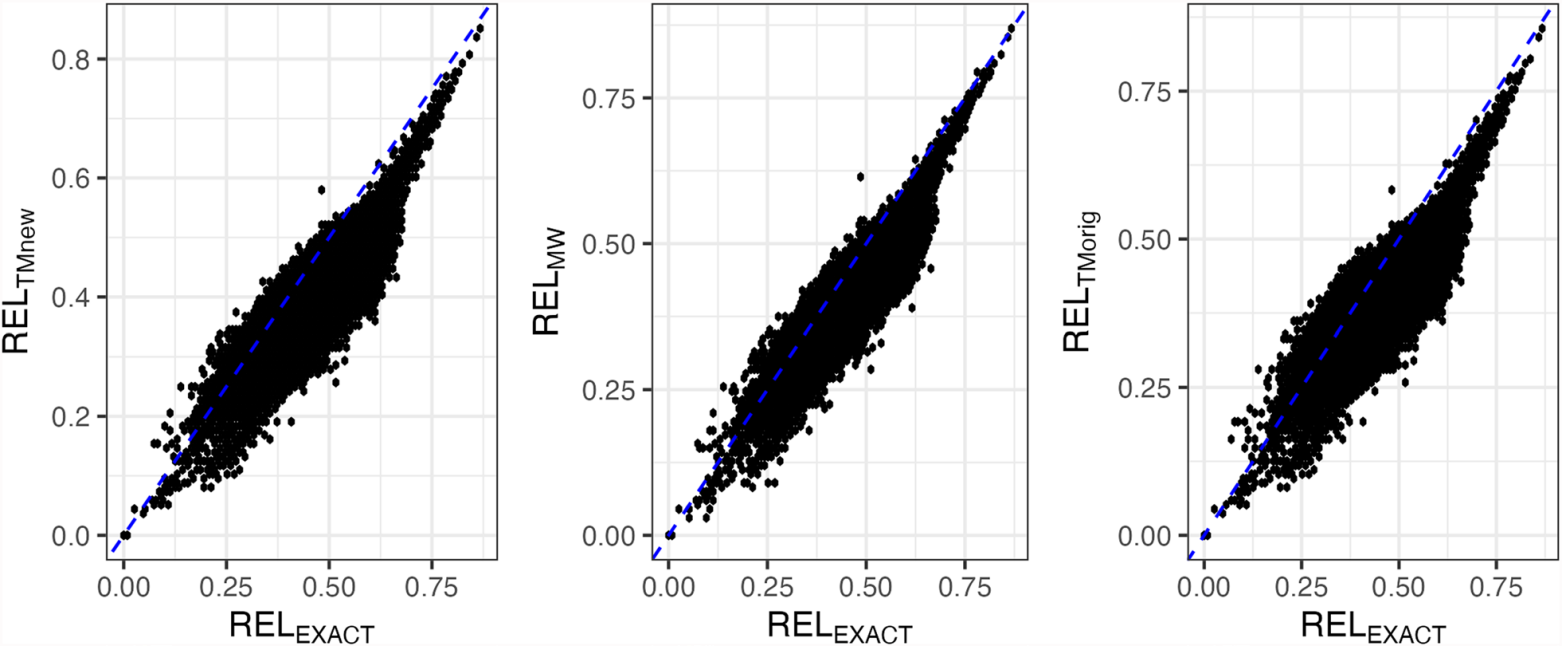
Reliabilities of cows with records from DataReduced. REL_EXACT_: Exact reliabilities from the inverse of the coefficient matrix with blupf90+; REL_MW_: Approximated reliabilities based on Misztal–Wiggans; REL_TMnew_: Approximated reliabilities based on modified Tier–Meyer; REL_TMorig_: Approximated reliabilities based on original Tier–Meyer

In DataReduced, we also studied cow reliabilities. Figure 5 shows the reliabilities of cows with records, indicating no impact of considering the HS effect on reliability estimates. Table 3 shows correlations exceeding 0.81 and regression slopes around 1 for $RE{L}_{MW}$, $RE{L}_{TMnew}$ and $RE{L}_{TMorig}$. The HS effect had a minimal impact on cows, suggesting that the effect of the sire’s bias in reliability does not affect its daughters.

**Table 3:** Correlation and regression of REL_EXACT_ with approximated reliabilities for cows with records in DataReduced

	Mean	b_1_	Correlation
REL_EXACT_^a^	0.50		
REL_MW_	0.45	1.07	0.91
REL_TMnew_	0.43	1.00	0.81
REL_TMorig_	0.43	1.02	0.81

^a^REL_EXACT_: Exact reliabilities from the inverse of the coefficient matrix with blupf90 + ; REL_MW_: Approximated reliabilities based on Misztal–Wiggans; REL_TMnew_: Approximated reliabilities based on modified Tier–Meyer; REL_TMorig_: Approximated reliabilities based on standard Tier–Meyer

**Fig. 6:**
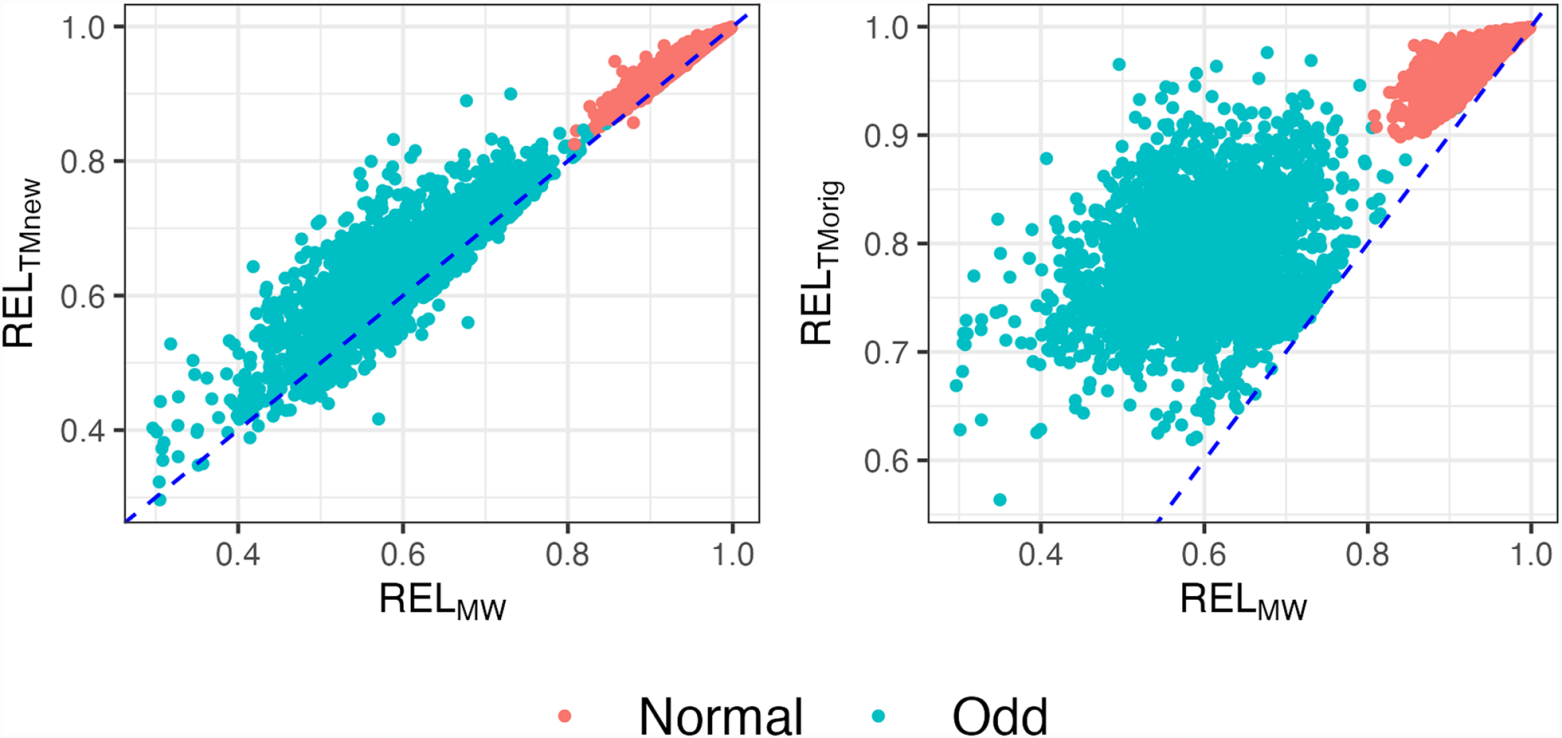
Reliabilities of Odd and Normal sires from DataFull. Odd: Sires with many daughters concentrated in a small number of herds; Normal: Sires with daughters more evenly distributed across many herds; REL_EXACT_: Exact reliabilities from the inverse of the coefficient matrix with blupf90+; REL_MW_: Approximated reliabilities based on Misztal–Wiggans; REL_TMnew_: Approximated reliabilities based on modified Tier–Meyer; REL_TMorig_: Approximated reliabilities based on original Tier–Meyer

In DataFull, we were unable to obtain $RE{L}_{EXACT}$. Based on previous results, we considered $RE{L}_{MW}$ as a good approximation to $RE{L}_{EXACT}$. Figure 6 and Table 4 compare $RE{L}_{TMnew}$ and $RE{L}_{TMorig}$ to $RE{L}_{MW}$ for both Odd and Normal sires in DataFull. The results show that $RE{L}_{TMnew}$ and $RE{L}_{MW}$ had similar means across both types of sires. The modified Tier–Meyer method, $RE{L}_{TMnew}$, had dispersions of 0.87 and 1.10 for Odd and Normal sires, respectively, but ignoring the HS in $RE{L}_{TMorig}$ had regression coefficients far from 1. The differences were more pronounced for Odd sires, with correlations for $RE{L}_{TMnew}$ of 0.85 with $RE{L}_{MW}$ and 0.45 for $RE{L}_{TMorig}$.

**Table 4:** Reliability means, correlation, and regression of REL_MW_ on approximated reliabilities REL_TMnew_ and REL_TMorig_ of Odd and Normal sires from DataFull

	Odd^a^			Normal		
	Mean	b_1_	Correlation	Mean	b_1_	Correlation
REL_MW_^b^	0.60			0.94		
REL_TMnew_	0.63	0.87	0.85	0.95	1.10	0.98
REL_TMorig_	0.78	0.45	0.31	0.97	1.41	0.85

^a^Odd: Sires with many daughters concentrated in a small number of herds (N = 2600); Normal: Sires with daughters more evenly distributed across many herds (N = 1950)

^b^REL_MW_: Approximated reliabilities based on Misztal–Wiggans; REL_TMnew_: Approximated reliabilities based on modified Tier–Meyer; REL_TMorig_: Approximated reliabilities based on standard Tier–Meyer

The bulls in our example had $RE{L}_{TMnew}$ and $RE{L}_{TMorig}$ of 0.71 and 0.97 (JEUSA000117723269) and 0.65 and 0.96 (JE840003012659218) in DataFull. Values for $RE{L}_{TMorig}$ agree with CDCB's traditional reliabilities. Correcting for the HS effect as in $RE{L}_{TMnew}$ likely reduced the overestimation, although we do not have $RE{L}_{EXACT}$ values to compare.

This study confirmed with both real and simulated data that ignoring the specifics of the HS effect in reliability approximation leads to overestimated reliabilities for sires with daughters concentrated in few herds, even if the number of daughters is large [2, 7, 13]. This could lead to incorrect selection decisions and different appraisals of risk for sires’ owners. In the past, the inclusion of HS in reliability approximation was highly emphasized [5, 8, 9], but then it had less importance due to the use of AI sires, and recently, this effect has regained importance for certain sires. Decades ago, Kelleher et al. [2] suggested that using AI sires across herds would make HS less relevant for reliability approximation. Here, we confirm that the effect is still relevant even with AI because some sires are not spread across herds.

Exact reliabilities are difficult to compute due to the computational demands of inverting the MME, so approximation methods are needed to calculate PEV. The method by Misztal and Wiggans [13] performed well, though its application to complex models (e.g., random regression) is not straightforward. We found that the more general method by Tier and Meyer [10] did not explicitly address the HS effect, resulting in overestimated reliabilities for sires. We modified the Tier–Meyer method by assigning a weight $\left(c\right)$ based on HS counts of records, to reduce the amount of information the cow transfers back to its sire. By doing so, we compensated for the overestimation of reliabilities, aligning estimates with the true values. With the modification, both methods (Misztal–Wiggans and modified Tier–Meyer) yielded similar results and close to exact values obtained by inversion.

## Conclusions

This study evaluated the bias in approximated reliabilities due to ignoring the HS effect in two approximated methods used for computing reliabilities. We identified excessively high approximated reliabilities, particularly when sires have daughters concentrated in a few herds, and the HS effect was not correctly accounted for. The existing Misztal–Wiggans algorithm for approximated reliabilities considers the HS well. We modified the Tier–Meyer method to weigh down the reliability of a sire, accounting for the loss of information due to the descendants of a sire being in a few HS classes. As a result, the modified Tier–Meyer algorithm performed well and eliminated the bias.

## Data Availability

The used datasets are the property of dairy producers who supplied the data through their participation in the Dairy Herd Improvement program.
